# Early-Stage Hydration and Product Evolution in Calcium Hydroxide-Activated Lithium Slag

**DOI:** 10.3390/gels12050359

**Published:** 2026-04-24

**Authors:** Baoliang Li, Liying Shi, Hongrui Shang, Wangzi Li, Shouhua Liu, Binbin Huo, Baizhan Ding, Guojun Huang

**Affiliations:** 1 Faculty of Architecture and Civil Engineering, Huaiyin Institute of Technology, Huai’an 223001, China; lbljndx@126.com (B.L.); sly18151607639@163.com (L.S.); shang_hongrui@126.com (H.S.); 18361264948@163.com (W.L.); 2 Jiangsu Key Laboratory of Construction Materials, School of Materials Science and Engineering, Southeast University, Nanjing 211189, China; huobinbin@cumt.edu.cn; 3 School of Mines, China University of Mining and Technology, Xuzhou 221116, China; 4 Huaian Construction Engineering Quality Testing Center Co., Ltd., Huai’an 223003, China; lwxs2005@163.com (B.D.); 13952384010@163.com (G.H.)

**Keywords:** lithium slag, calcium hydroxide, hydration process, hydration products

## Abstract

This study used calcium hydroxide (CH) to simulate the alkaline environment of cement and to activate lithium slag (LS), aiming to reveal the mechanism of LS in cement. The early-age hydration of LS blended with 10 wt.% CH was monitored via isothermal calorimetry (ICC) at ambient temperature, followed by a comparative analysis of phase assemblage, microstructure, and macroscopic properties under standard and steam curing conditions. The results show that LS exhibits superior early reactivity within the first 9 h, which is attributed to abundant ettringite formation. Two distinct exothermic peaks were identified during LS-CH hydration, corresponding to (i) ettringite formation accompanied by LS dissolution and C–S–H precipitation, and (ii) CaCO_3_ crystallization and renewed ettringite formation. The hydrated paste consists of abundant AFt, CaCO_3_ polymorphs, unreacted LS particles, and a small amount of C–S–H gel with a low Ca/Si ratio and incorporating Al and S. This unique phase assemblage results in a coarser pore structure and lower specific surface area compared with conventional cement paste. Nevertheless, the system achieves a relatively high 28-day compressive strength, highlighting the promise of LS-CH blends as sustainable cementitious materials.

## 1. Introduction

Lithium slag (LS) is a byproduct generated from lithium carbonate production via the sulfuric acid method [1], and its accumulation is directly linked to the rapid expansion of the lithium battery industry. In China, the remarkable growth of the new energy vehicle sector has led to a rapid increase in lithium salt production, inevitably resulting in a steady rise in LS emissions, which currently amount to approximately 6 million tons annually [2,3]. This massive accumulation not only occupies farmland but also poses a significant threat to the environment.

Previous studies have shown that the mineral composition of LS includes leached spodumene, quartz, gypsum, and carbonate [4]; and its chemical composition is dominated by SiO_2_ (48.6~63.1%), Al_2_O_3_ (14.0~20.7%), and SO_3_ (4.5~9.3%), along with small amounts of CaO (3.6~12.1%) [5,6,7]. Although LS contains high levels of SiO_2_ and Al_2_O_3_, these components are primarily present in the form of leached spodumene rather than in an amorphous phase [8].

The high Al_2_O_3_ and SiO_2_ contents in lithium slag enable its use in preparing zeolite X and ceramic glazed tiles [6,9]. On the other hand, its high gypsum content makes it a high-quality raw material for producing gypsum products. In addition, Ding et al. [10] noted that LS is effective in mitigating expansion induced by the alkali-silica reaction (ASR). However, incorporating LS powder into concrete may lead to micro-expansion due to ettringite formation. Therefore, this micro-expansion should be isolated when evaluating the ASR-inhibiting effect of LS powder. He et al. [11] demonstrated that mine filling materials with excellent unconfined compressive strength can be prepared using cement, lithium slag, and fly ash at a mass ratio of 2:1:1. They further reported that the addition of NaOH can improve the pozzolanic activity of lithium slag in mine backfill binders [12]. Furthermore, LS has been used as a white pigment [13,14] and in the production of lightweight aggregates [15]. Despite its applications in various fields, the utilization rate of lithium slag remains low. Therefore, exploring efficient methods to improve the utilization of LS is of great significance.

In recent years, the use of supplementary cementitious materials (SCMs) in concrete has gained increasing attention owing to their economic benefits, environmental advantages, and technological improvements [16]. LS contains large amounts of SiO_2_ and Al_2_O_3_, which impart pozzolanic activity; thus, it also represents a promising candidate for use as an SCM [17]. However, like other SCMs, LS hinders the early strength development rate of cementitious systems. Zhang [5] reported that a higher LS content leads to greater deterioration in the early strength of concrete. To improve the reactivity of LS, Liu combined alkali activation with thermal treatment, using this method, the 1-day compressive strength of alkali-activated LS was increased from 12.8 MPa to 47.7 MPa [18]. Tan et al. [19,20] found that wet-grinding yields much finer LS particles than dry-grinding, thereby accelerating the formation of hydration products in concrete incorporating LS as an SCM. Previous work by the authors demonstrated that steam curing can significantly improve the demolding strength of LS-blended cement mortar [21,22]. However, the above-mentioned activation methods have issues in terms of environmental friendliness, energy consumption, and cost. Specifically, the strong alkali activation method not only causes severe corrosion to equipment but also increases construction operation risks and material costs. Meanwhile, the drying step after wet-grinding and the steam curing process incur high energy costs.

Although the incorporation of LS can hinder early strength development, it enhances the early crack resistance of concrete by forming more fibrous ettringite [23]. Moreover, when the replacement level is below 20%, LS can improve the long-term (≥28 d) compressive strength of concrete. Li et al. [8,24] reported that incorporating LS can enhance the durability of concrete, including sulfate resistance, elastic modulus, drying shrinkage, and creep behavior. He et al. [25] showed that replacing silica fume with LS can increase the hydration degree of ultra-high-performance concrete. However, at high LS replacement ratios, the high gypsum content in LS may lead to a risk of delayed ettringite formation in concrete. Notably, early-age steam curing has been proven effective in addressing this issue [8].

Not surprisingly, the incorporation of LS reduces the Ca/Si ratio of C–S–H gels. Meanwhile, our study identified the presence of equant grain-shaped C–S–H gels and cubic CaCO_3_ in lithium slag-blended cement [22]. Beyond these observations, little is known about the characteristics of hydration products in lithium slag-blended cement. Furthermore, the influence of the high sulfate and carbonate contents in LS on cement hydration remains unclear.

Calcium hydroxide (CH) is one of the main hydration products in cement and the primary reactant for SCMs in concrete. Therefore, to explore the hydration mechanism of SCMs in concrete, CH is often used to simulate the alkaline environment in Portland cement pastes. This approach allows the direct investigation of the reaction between CH and SCMs, providing insight into the hydration process and products of SCMs in concrete. However, unlike common SCMs such as ground granulated blast-furnace slag (GBFS) and fly ash (FA), LS contains carbonates and a higher SO_3_ content. The reaction mechanism between lithium slag and CH remains unclear.

Thus, this study first investigates the hydration process of LS blended with 10 wt.% Ca(OH)_2_ (LS-CH) using isothermal conduction calorimetry (ICC) and X-ray diffraction (XRD). It then characterizes the hydration products of LS-CH pastes under standard curing conditions using XRD, scanning electron microscopy with energy-dispersive spectroscopy (SEM–EDS), thermogravimetric analysis (TG–DTG), Fourier transform infrared spectroscopy (FTIR), and Brunauer–Emmett–Teller (BET) analysis, while their mechanical properties are evaluated through strength tests. Given the increasing adoption of precast concrete construction, this study further examines the hydration products and mechanical properties of LS-CH pastes under steam curing.

Based on the experimental investigation, this study provides a comprehensive understanding of the hydration behavior of the LS-CH system, with detailed descriptions of its microstructural evolution and phase development. These findings clarify the role of lithium slag in cementitious matrices and validate its potential as a sustainable, low-carbon construction material.

## 2. Results and Discussion

### 2.1. Results of Material Characterization

**Figure 1:**
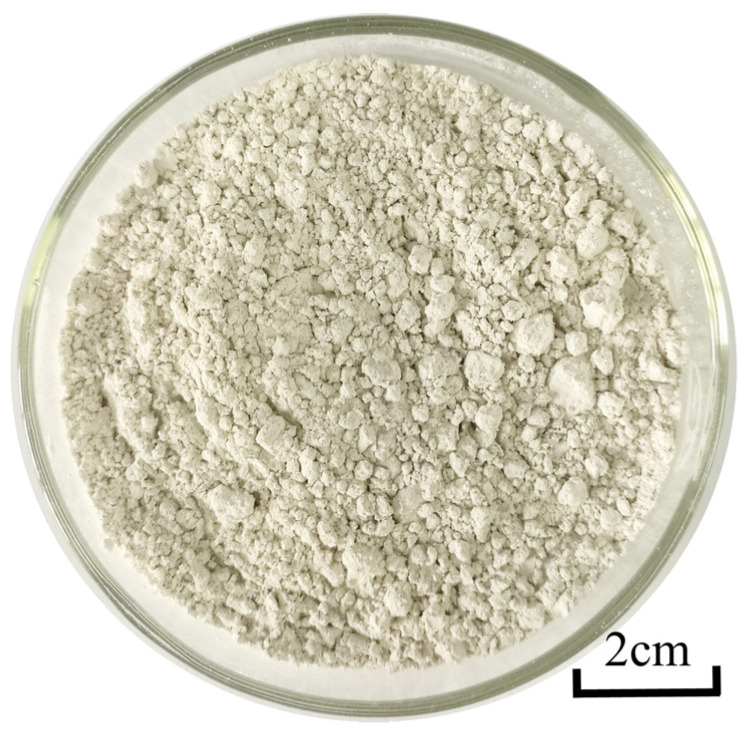
Appearance of LS powder.

As shown in Figure 1, the lithium slag (LS) powder was yellowish-white in appearance. Its chemical composition, analyzed by X-ray fluorescence (XRF), is presented in Table 1. Like ground granulated blast-furnace slag (GBFS) and fly ash (FA), LS is rich in SiO_2_ and Al_2_O_3_, with the main distinction being its higher SO_3_ content. SiO_2_, Al_2_O_3_, and SO_3_ together account for 89.03 wt.% of LS by mass. Other minor components include CaO, P_2_O_5_, K_2_O, and Fe_2_O_3_. Notably, the CaO content in LS is relatively low, even lower than that in FA. Accordingly, based on the data in Table 1, the molar Ca/Si ratio of LS is merely 0.08.

LS has a density of approximately 2.50 g/cm^3^, which is comparable to that of FA. For a LS-water suspension with a water-to-LS mass ratio of 10:1, the pH value reached 7.5 after 4 h of mixing, whereas that of tap water was 7.9. Therefore, LS can be classified as an acidic slag.

**Figure 2:**
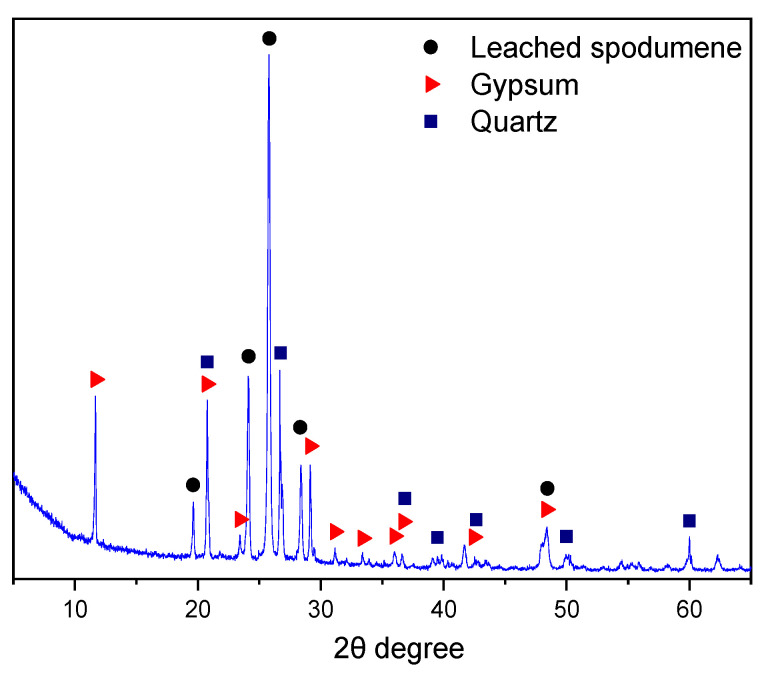
X-ray diffraction pattern of LS.

The XRD results (Figure 2) indicated that LS was primarily composed of 7.9% quartz (SiO_2_), 66.2% leached spodumene (LiAlSi_2_O_6_), 13.3% gypsum (CaSO_4_·2H_2_O), and 12.6% amorphous phase, as calculated via the XRD/Rietveld method.

**Figure 3:**
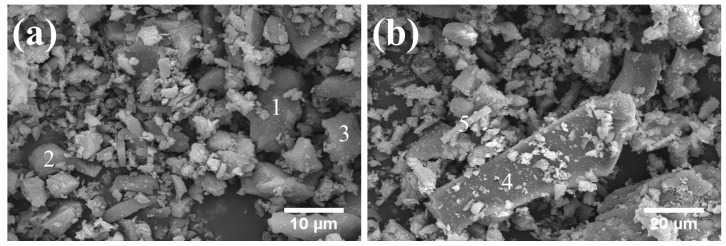
The morphology of LS under SEM: (**a**) morphology of LiAlSi_2_O_6_; (**b**) morphology of CaSO_4_·2H_2_O.

Figure 3 illustrates the morphology of LS particles, which exhibit an angular, distinct crystalline structure. The atomic composition of individual particles, determined by EDS results, is listed in Table 2. Smaller particles (Particles 1, 2, and 3) were predominantly LiAlSi_2_O_6_, whereas larger particles (Particles 4 and 5) were mainly CaSO_4_·2H_2_O. This can be attributed to the fact that LiAlSi_2_O_6_ is readily crushed during grinding [26], while gypsum tends to agglomerate owing to its higher moisture content and viscosity.

**Figure 4:**
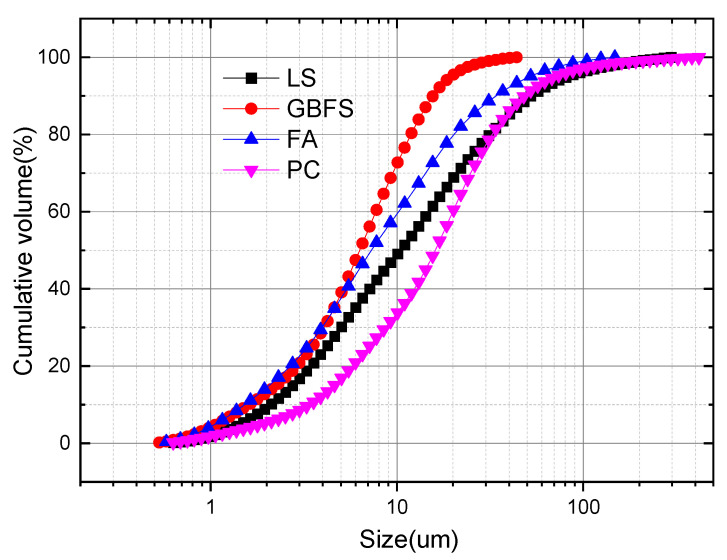
Particle size distribution of LS powder.

Figure 4 presents the particle size distribution of LS powder, measured using a laser particle size analyzer. The particle size of LS powder ranged from 0.6 μm to 296 μm, with an average particle size of 10.45 μm. This value was slightly larger than those of FA (7.43 μm) and GBFS (GBFS, 6.33 μm) used in this study, yet smaller than that of PC (16.70 μm).

**Figure 5:**
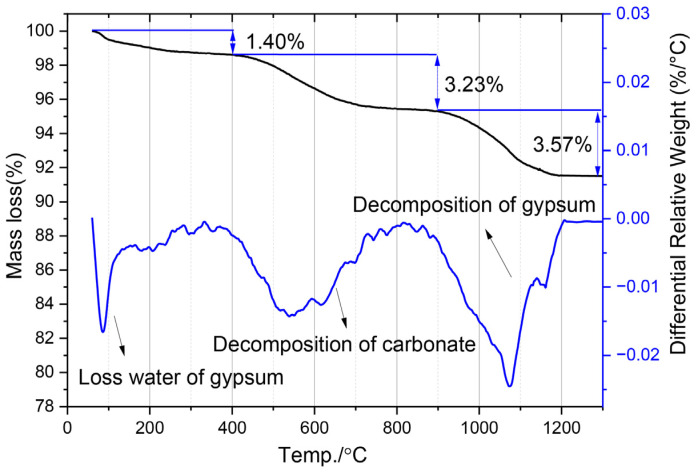
TG/DTG curves of LS powder.

Thermogravimetric (TG/DTG) analysis (Figure 5) indicated that, in addition to gypsum, lithium slag contained carbonates, with a loss on ignition of 8.2% below 1300 °C [27,28,29]. The high carbonate content in LS mainly exists in the forms of CaCO_3_, Na_2_CO_3_, and Li_2_CO_3_, whereas conventional SCMs contain very little carbonate, especially Na_2_CO_3_ and Li_2_CO_3_.

**Figure 6:**
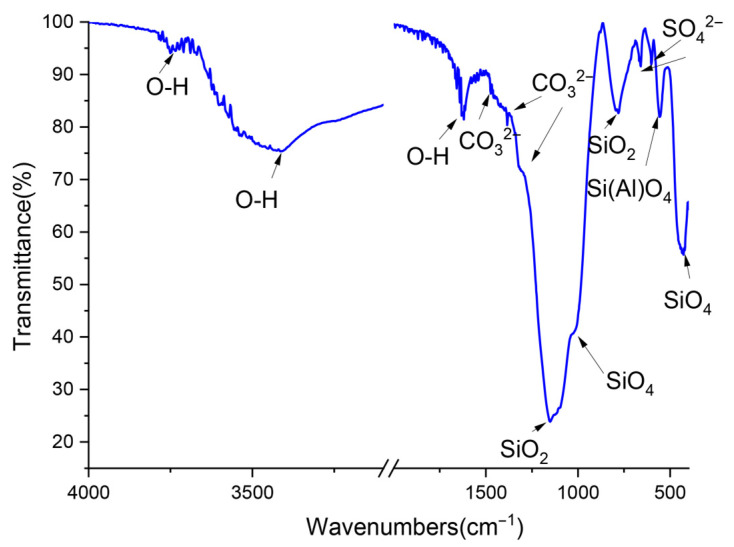
FTIR result of LS powder.

The FTIR results (Figure 6) further confirmed the presence of sulfates and carbonates in lithium slag. As reported in our previous study [8,22], these sulfates mainly exist in the forms of gypsum and sodium sulfate.

The 28-day pozzolanic activity of LS was 92%, which was slightly lower than that of GBFS (98%) but higher than that of FA (76%).

### 2.2. Hydration Heat Evolution of LS-CH Pastes

**Figure 7:**
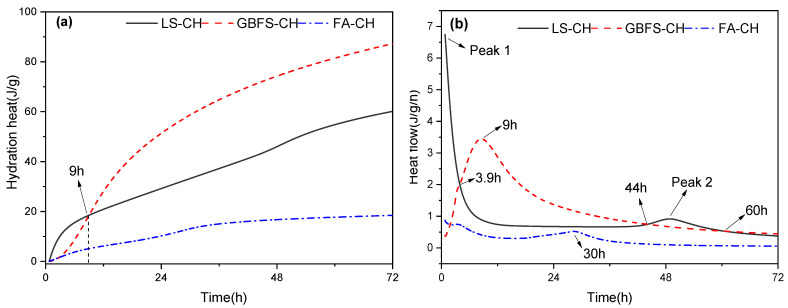
Isothermal calorimetry results of LS-CH pastes: (**a**) cumulative hydration heat, (**b**) heat flow.

The cumulative hydration heat and heat flow of LS-CH pastes with a water-to-binder ratio of 0.3 at 20 °C are presented in Figure 7. To clarify the hydration process of LS, the hydration heat results of GBFS-CH and FA-CH (prepared with the same mix proportion as LS-CH) are also included in Figure 7. As observed, the 72 h cumulative hydration heat of GBFS-CH was the highest at 87.2 J/g, followed by LS-CH at 60.30 J/g, while FA-CH exhibited the lowest value at 18.51 J/g. This indicates that the reactivity of LS is significantly higher than that of FA but slightly lower than that of GBFS within 72 h. However, it is noteworthy that the cumulative hydration heat of LS-CH during the first 9 h was the highest, demonstrating that LS possesses the highest hydration reactivity in this early period.

FA is generally regarded as an inert material at very early ages, particularly within the 0–24 h period, and its influence on cement hydration is primarily attributed to the filling effect and the provision of nucleation and precipitation sites for C–S–H gels [30]. Consequently, the hydration exothermic rate of the FA-CH mixture fell below 1 J/(g·h), with only several minor exothermic peaks observed within 30 h, as depicted in Figure 7b. In contrast to FA-CH, both GBFS-CH and LS-CH exhibited relatively high hydration exothermic rates, especially within the first 24 h. Furthermore, the hydration process of the GBFS-CH paste featured a significantly larger and broader exothermic peak, which initiated at approximately 0–1 h and terminated at roughly 48 h. This phenomenon is likely due to the formation of C–S–H gels [31].

However, two distinct exothermic peaks were observed during the hydration heat release of LS-CH pastes. The first exothermic peak was significantly higher than those of both GBFS-CH and FA-CH, whereas the second exothermic peak was relatively small: it was similar to that of FA-CH at approximately 30 h, but much lower than that of GBFS-CH at around 9 h. Notably, the hydration exothermic rate of LS-CH exceeded that of GBFS-CH again at 44 h. Thereafter, the two curves followed a similar trend after 60 h of hydration.

**Figure 8:**
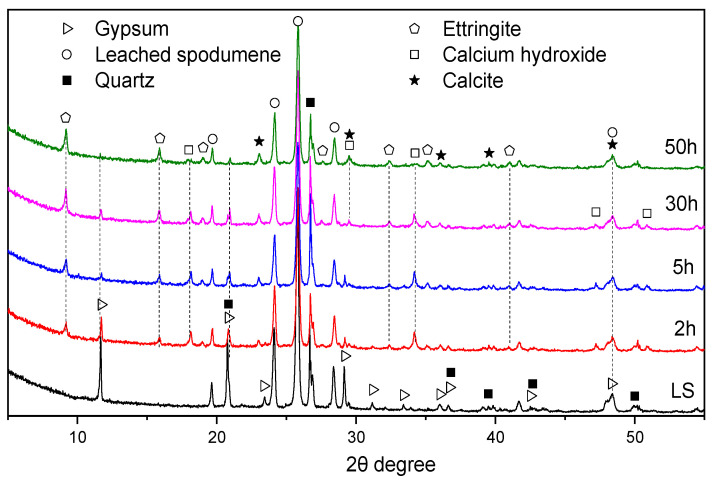
XRD pattern of LS-CH pastes hydrated from 2 h to 50 h.

To further investigate the underlying causes of the two exothermic peaks formed during the hydration of LS-CH paste, XRD analysis was performed on its hydration products cured at 20 °C for 2 h, 5 h, 40 h, and 50 h in a sealed centrifuge tube. The XRD patterns were collected over a 2θ range of 5° to 55°, with the results presented in Figure 8.

As shown in Figure 8, aside from AFt, no obvious crystalline phases were generated in the LS-CH paste between 2 h and 5 h. This suggests that the first exothermic peak in Figure 7b is primarily attributed to the formation of ettringite, together with the heat released from wetting and dissolution. Notably, the final setting time of the LS-CH paste was determined to be approximately 2 h. This indicates that the C–S–H gels formed by the reaction between LS and CH within 2–5 h further contributed to the hydration heat corresponding to the first exothermic peak.

As illustrated in Figure 7b, a comparison of the hydration heat flow curves of LS-CH and GBFS-CH before 3.9 h shows that, despite GBFS having a higher amorphous phase content than LS, the heat flow of the GBFS-CH system during this period is considerably lower than that of LS-CH. Based on the above analysis, the heat released by GBFS-CH before 3.9 h is mainly attributable to C–S–H formation. Given the lower amorphous content in lithium slag, the thermal contribution from C–S–H formation in the LS-CH system would be correspondingly smaller at the same hydration age. It can therefore be inferred that C–S–H formation is not the primary mechanism responsible for the first exothermic peak observed in LS-CH. In contrast, LS contains a substantially higher SO_3_ content. These results suggest that the first exothermic peak in the LS-CH system arises mainly from ettringite formation, with only a limited contribution from C–S–H gels. Accordingly, the enhanced hydration activity of lithium slag within the first 9 h can be attributed primarily to ettringite formation.

Between 30 h and 50 h, CaCO_3_ was detected, while the diffraction peak of gypsum decreased further. This suggests that the second exothermic peak can be attributed to the reformation of AFt, along with the formation of CaCO_3_. The formation of AFt between 30 h and 50 h is likely related to the renewed dissolution of Al_2_O_3_, which is similar to the corresponding process during cement hydration [32]. Furthermore, the presence of residual unreacted gypsum in the LS-CH mixture after 50 h of hydration indicates that the second broad exothermic peak in Figure 7b does not correspond to gypsum depletion or AFm formation [33,34].

### 2.3. Hydration Products of LS-CH Pastes Determined by XRD

#### 2.3.1. Hydration Products of LS-CH Pastes Under Normal Curing Condition

**Figure 9:**
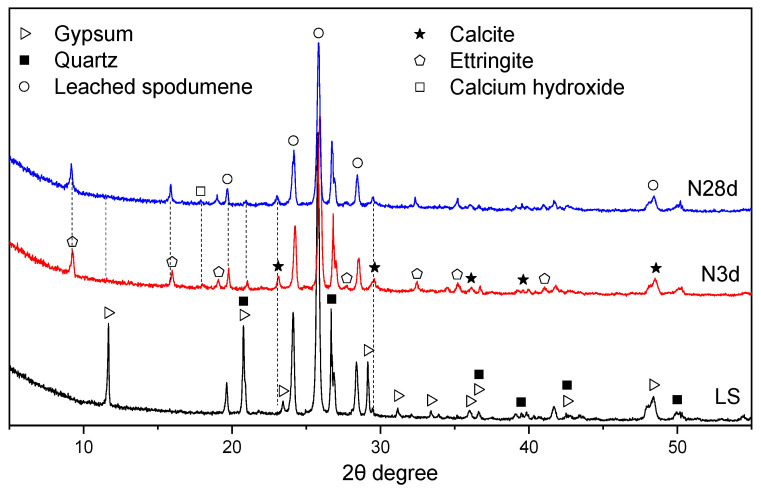
XRD pattern of LS-CH pastes hydrated at 3 d and 28 d under normal curing conditions.

**Figure 10:**
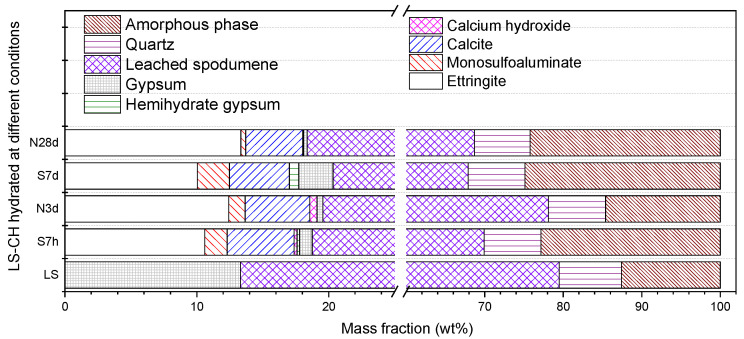
The content of phases in LS-CH pastes derived from XRD/Rietveld methods, wt.%.

Figure 9 shows the XRD patterns of LS-CH pastes cured for 3 days (N3d) and 28 days (N28d) under normal curing conditions. Figure 10 presents the relative content of each phase in LS-CH pastes, as determined by XRD/Rietveld analysis.

The results show that the diffraction peaks of gypsum (originally from LS) gradually weakened over time, whereas those of quartz remained largely unchanged. This indicates that the quartz in LS is either inert or exhibits extremely low reactivity. Notably, the main diffraction peak of LiAlSi_2_O_6_ slightly decreased over time, suggesting that, in addition to amorphous phases, LiAlSi_2_O_6_ can also slowly participate in the hydration reaction. Previous studies have identified leached LiAlSi_2_O_6_ as a zeolite-like phase [26] with relatively low pozzolanic activity, comparable only to that of FA [35].

At 3 d and 28 d, the primary reactive components in LS consisted of LiAlSi_2_O_6_ along with amorphous Al_2_O_3_, SiO_2_, and crystalline CaSO_4_·2H_2_O (gypsum). The major hydration products in LS-CH pastes included C–S–H gels, ettringite (AFt), and CaCO_3_ (calcite). Notably, owing to their amorphous nature, C–S–H gels could not be detected by XRD analysis, whereas the crystalline phases (AFt and CaCO_3_) were clearly identified. It should be noted that the formation of CaCO_3_ arose not only from the reaction between CH and carbonate but also from the carbonation of CH during specimen preparation. Accordingly, the primary phases in hardened LS-CH pastes were unreacted CH, C–S–H gels, AFt, CaCO_3_, SiO_2_ (quartz), and LiAlSi_2_O_6_.

Similar to metakaolin [36,37,38], the main chemical reaction in LS-CH pastes involves the reaction between amorphous Al_2_O_3_, SiO_2_, and CH. However, LS contains considerable amounts of SO_3_ (primarily present as gypsum and other sulfates) and carbonates (Figure 5). Meanwhile, LiAlSi_2_O_6_, which is rich in Al_2_O_3_ and SiO_2_, can also participate in hydration reactions, although its reactivity is relatively low. Consequently, the overall hydration process can be described by reactions (1)–(4).

The formation of C–S–H gels:

(1) $$SiO_{2}+Ca{\left(OH\right)}_{2}+H_{2}O\to C–S–H$$

The formation of AFt:

(2) $$Al_{2}O_{3}+SO_{3}+Ca{\left(OH\right)}_{2}+H_{2}O\to C_{6}A{\bar{S}}_{3}H_{32}(AFt)$$

The formation of CaCO_3_ [39]:

(3) $${CO_{3}}^{2-}+Ca^{2+}\to CaCO_{3}$$

(4) $$Ca{\left(OH\right)}_{2}+CO_{2}\to CaCO_{3}+H_{2}O$$

The reaction between LS and CH solution proceeds via a mechanism analogous to the pozzolanic reaction of LS in cement systems. The high-pH CH solution actively disrupts Si-O and Al-O bonds, promoting the formation of Al-bearing C–S–H gels via the reaction of SiO_2_ and Al_2_O_3_ (from both amorphous phases and partial dissolution of LiAlSi_2_O_6_) with CH. The proposed reaction mechanism comprises three key steps:

(1) CH-activated dissolution of SiO_2_ from LS releases SiO_4_^4−^ species into solution;
(2) the subsequent reaction of these silicate ions with CH forms C–S–H gels [40,41];
(3) the concurrent incorporation of aluminum species (released from Al_2_O_3_ dissolution) into the C–S–H structure yields C–(A)–S–H gels [42].

Simultaneously, in the presence of gypsum and other sulfates, dissolved Al_2_O_3_ reacts with CH to initially form AFm phases, which subsequently convert to ettringite (AFt). Notably, carbonate ions may partially or fully replace sulfate in the ettringite structure through anion substitution [40]. Furthermore, carbonate species derived from LS can react directly with CH in aqueous conditions to precipitate CaCO_3_ (calcite). Additionally, CaCO_3_ may form indirectly through the carbonation of CH upon exposure to atmospheric CO_2_.

Unlike GBFS, LS contains both amorphous and crystalline phases. The crystalline phases in LS (e.g., gypsum and carbonates) mainly contribute to its early hydration activity within 9 h, as evidenced by the significantly higher hydration heat of the LS-CH system relative to GBFS-CH in Figure 7. In contrast, the amorphous phase and spodumene in LS are primarily responsible for its later-stage reactivity. In the late hydration stage, spodumene and the amorphous phase in LS can react continuously with CH, releasing hydration heat steadily. Nevertheless, the performance is limited by several factors: the reactivity of spodumene is only comparable to that of FA; LS does not undergo the high-temperature water quenching process used for GBFS, leading to lower activity of its amorphous phase; and the amorphous phase content in LS is considerably lower than in GBFS. Owing to the combined effect of these factors, the hydration heat release of the LS-CH system after 9 h is lower than that of GBFS-CH.

#### 2.3.2. Hydration Products of LS-CH Pastes Under Steam Curing Condition

**Figure 11:**
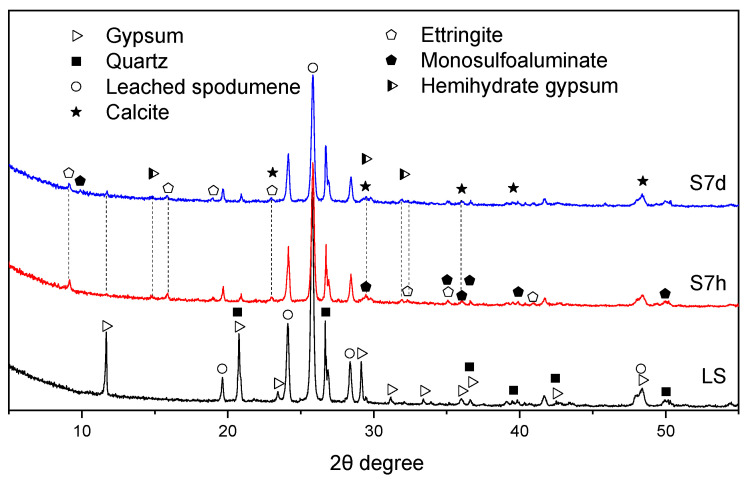
XRD pattern of LS-CH pastes hydrated at 7 h and 7 d under 80 °C steam curing.

As steam curing is widely used in precast concrete production, this study also investigated the hydration products of LS-CH paste under steam-cured conditions. Figure 11 shows the XRD patterns of LS-CH pastes cured at 80 °C for 7 h (S7h) and 7 days (S7d) under steam curing. Analysis indicated that the main hydration products were C–S–H gels, ettringite (AFt), monosulfoaluminate (AFm), bassanite (CaSO_4_·0.5H_2_O), and calcite (CaCO_3_), together with unreacted LiAlSi_2_O_6_ and quartz. A comparison of Figure 10 and Figure 11 demonstrated that steam curing significantly accelerates the hydration rate of LiAlSi_2_O_6_ compared with standard curing conditions, which is consistent with previous results [8].

Taylor et al. [43] demonstrated that excess gypsum can stabilize ettringite (AFt) even under elevated temperature, which explains its persistence after 7 h and 7 d of steam curing in this study. However, given the well-documented thermal instability of AFt at 80 °C [44], we observed its gradual conversion to monosulfoaluminate (AFm) and bassanite (CaSO_4_·0.5H_2_O) after the initial 7 h of curing, a finding consistent with previous reports [45]. Concurrently, elevated temperatures also accelerated the decomposition of gypsum into bassanite [27].

With prolonged steam curing (7 d), two distinct phenomena were observed:

(1) further decomposition of AFt, as indicated by the progressive weakening of its characteristic XRD peaks (Figure 11);
(2) the reappearance of gypsum diffraction signals.

This latter observation suggests the existence of a dynamic equilibrium involving sulfate release from continuous AFt decomposition and subsequent rehydration of bassanite back to gypsum under the steam curing conditions.

### 2.4. Morphology of Hydration Products in LS-CH Pastes Under SEM

#### 2.4.1. LS-CH Pastes After 28 Days of Standard Curing

**Figure 12:**
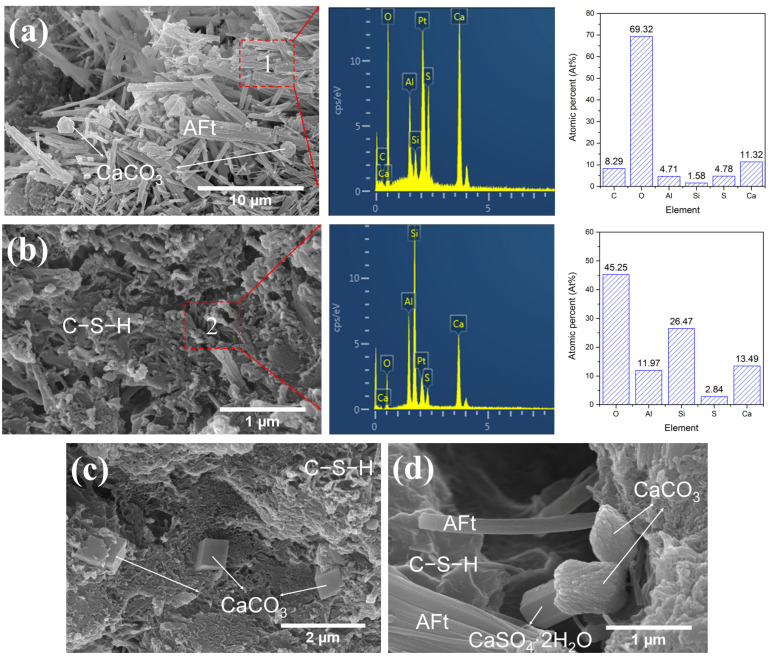
Morphology and composition of hydration products in N28d samples under SEM-EDS, (**a**) spherical CaCO_3_, needle-like AFt and its composition; (**b**) reticular C–S–H gels and EDS results of C–S–H; (**c**) cubic CaCO_3_; (**d**) prismatic gypsum and ellipsoid-like CaCO_3_.

Given the high SO_3_ content in LS, SEM-EDS was used to conduct an in-depth investigation into the morphology and composition of hydration products in LS-CH pastes. The representative SEM-EDS results are presented below. Since no significant differences were observed in the hydration products of LS-CH pastes cured for 3 and 28 days, Figure 12 only presents the morphology and EDS results of hydration products in the 28-day LS-CH paste.

As shown in Figure 12, the hardened LS-CH paste consisted of six types of hydration products along with unreacted LS particles. These six hydration products were needle-like AFt with a length of 1~10 μm (Figure 12a,d), spherical CaCO_3_ with a particle size of approximately 1 μm (Figure 12a), porous reticular C–S–H (Figure 12b,d), cubic CaCO_3_ with a particle size of about 1 μm (Figure 12c), ellipsoidal CaCO_3_ with a particle size of approximately 2 μm (Figure 12d), and hexagonal prismatic gypsum with a particle size of approximately 1 μm (Figure 12d).

Ettringite, which contains 32 bound water molecules, has a large molar volume that contributes to an increased solid volume and reduced porosity in hardened pastes [46]. However, when formed in excess, the generation of loosely packed, polydisperse ettringite needles (Figure 12a) can reduce the packing density and hinder the densification of the LS-CH matrix. Additionally, the high carbon content in the EDS analysis of ettringite (Figure 12a) suggests that carbonates from the lithium slag were involved in its formation.

It is well established that CaCO_3_ crystals typically exhibit three distinct morphologies: cubic calcite, spherical vaterite, and needle-like aragonite. Among these, calcite is the most thermodynamically stable under ambient conditions, whereas vaterite and aragonite tend to convert to calcite over time [47]. Furthermore, the morphology of CaCO_3_ is highly dependent on precipitation conditions, including solution pH, temperature, the presence of foreign ions (e.g., Mg^2+^) and organic additives, as well as the degree of supersaturation [47,48].

In this study, the low alkalinity of the LS-CH hardened paste, carbonates present in LS, elevated curing temperature, and the use of acetone to halt hydration are believed to contribute to the formation of multiple CaCO_3_ morphologies. Notably, CO_3_^2−^ and Ca^2+^ initially react to form metastable spherical or ellipsoidal vaterite (Figure 12d), which subsequently undergoes gradual transformation into calcite. This indicated that ellipsoidal vaterite acts as an intermediate phase during calcite formation under the given conditions.

Additionally, the presence of unreacted gypsum (Figure 12d) after 28 days of standard curing suggests incomplete sulfate consumption in the LS-CH paste. Consequently, special attention should be paid to the risk of delayed ettringite formation (DEF) when lithium slag is used as an SCM in concrete applications.

#### 2.4.2. LS-CH Pastes Steam-Cured for 7 h and 7 Days

**Figure 13:**
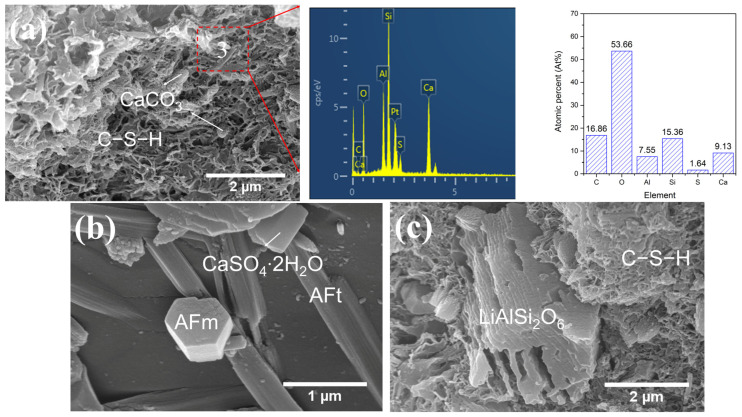
Morphologies and composition of hydration products in S7h samples under SEM-EDS, (**a**) reticular C–S–H gels and EDS results of C–S–H; (**b**) hexagonal plate-like AFm and prismatic gypsum; (**c**) layered LiAlSi_2_O_6_.

**Figure 14:**
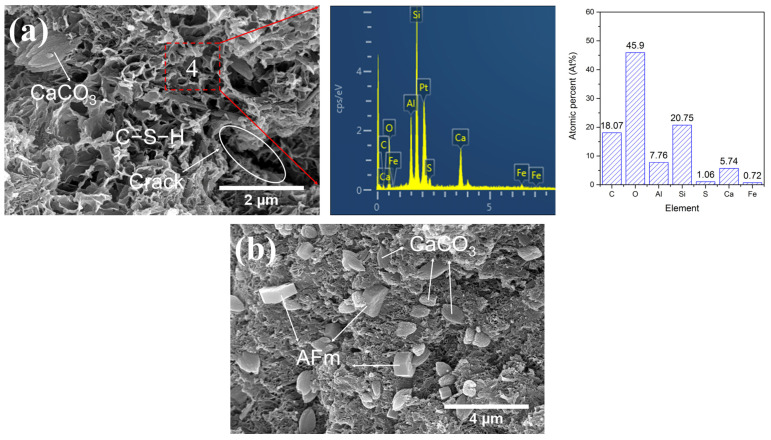
Morphologies and composition of hydration products in S7d samples under SEM-EDS, (**a**) ellipsoid-like CaCO_3_; reticular C–S–H gels and EDS results of C–S–H; (**b**) hexagonal plate-like AFm and ellipsoid-like CaCO_3_.

Figure 13 and Figure 14 present the microstructural characteristics of hydration products in LS-CH pastes after steam curing for 7 h and 7 days, respectively. The main hydration phases formed under steam curing include: (1) loose reticular C–S–H gels (Figure 13a,c and Figure 14a); (2) fibrous AFt with a length of 5 μm and a diameter of 0.5 μm (Figure 13b); (3) hexagonal plate-like AFm with a size close to 1 μm (Figure 13b and Figure 14b); (4) ellipsoidal CaCO_3_ with a particle size of 0.5~1 μm (Figure 13a and Figure 14b); and (5) short prismatic gypsum crystals with a particle size close to 1 μm (Figure 13b). Notably, the appearance of partially eroded LiAlSi_2_O_6_ after heat treatment indicates its involvement in the hydration reaction, especially at elevated temperatures. This observation is in good agreement with the XRD results.

Consistent with the high SiO_2_, Al_2_O_3_, and SO_3_ contents in lithium slag, the C–S–H gels in the LS-CH paste exhibited lower Ca/Si ratios (0.28~0.59) and higher Al/Si ratios (0.37~0.49) (Figure 12b, Figure 13a and Figure 14a), along with significant sulfate adsorption (Figure 12b, Figure 13a and Figure 14a). Previous studies have established that sulfate primarily adsorbs onto Si-O sites as CaSO_3_ complexes without being incorporated into the C–S–H gel structure [46,49], this adsorption behavior may subsequently promote the formation of AFt and AFm phases.

Interestingly, the C–S–H gels maintained a relatively loose, highly porous structure (Figure 12b, Figure 13a and Figure 14a) regardless of curing conditions. This observation aligns with the existing literature: Bérodier et al. [49] reported that elevated sulfate content reduces the bulk volume of C–S–H gels while increasing capillary porosity, whereas Adu-Amankwah et al. [50] observed that excess sulfate lowers gel water content and increases porosity. Although our results support these findings, the underlying mechanisms warrant further investigation.

### 2.5. TG/DTG Analysis Results of Hydration Products in LS-CH Pastes

**Figure 15:**
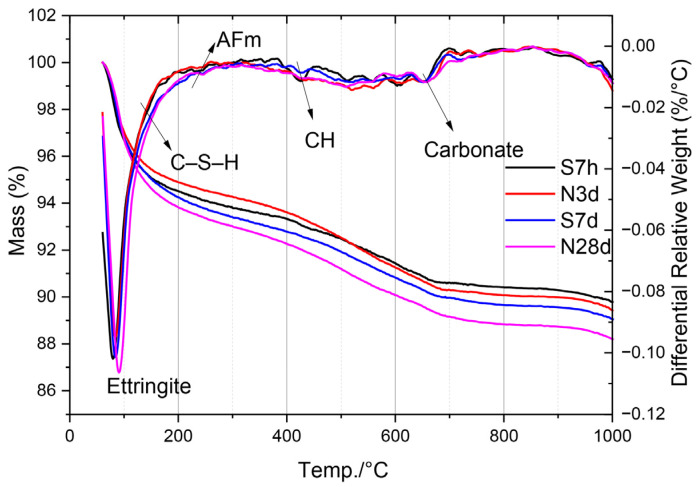
TG/DTG results of LS-CH pastes.

Figure 15 presents the TG/DTG results of LS-CH pastes under different curing conditions. The characteristic mass loss events within specific temperature ranges, along with their corresponding chemical reactions, are systematically summarized in Table 3. In addition, Table 4 provides a quantitative analysis of total weight loss percentages within defined temperature intervals for all investigated LS-CH paste samples.

The mass loss observed between room temperature and 400 °C primarily corresponds to the decomposition of newly formed hydration products in LS-CH paste, thus serving as a reliable indicator of hydration degree. Based on this analysis, the relative hydration degrees under different curing conditions followed the order: N28d > S7d > S7h > N3d. Although steam curing at early ages promotes hydration, prolonged steam curing (7 days) appeared to inhibit further hydration development. This phenomenon may be attributed to the gradual decomposition of ettringite under extended thermal treatment, as well as the limited CH content in the LS-CH mixture.

In addition, trace carbonate phases were detected in all four LS-CH paste samples. These carbonates may originate from: (1) reactions between inherent carbonates in LS and CH, (2) carbonation occurring during sample preparation or storage, or (3) residual unreacted carbonates in the original LS material.

Besides the non-evaporable water, the mass loss of the LS-CH paste between room temperature and 1000 °C also reflects the decomposition of carbonates, unreacted gypsum, and residual CH. Nevertheless, the total mass loss remained considerably lower than that of pure cement paste [8], indicating that the hydration degree of LS-CH pastes is relatively low compared with conventional cement systems.

### 2.6. Hydration Products of LS-CH Pastes Determined by FTIR

**Figure 16:**
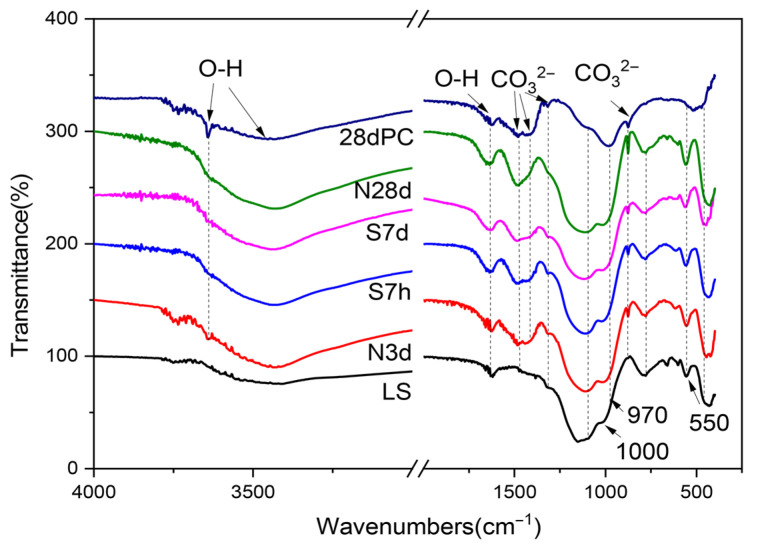
FTIR results of LS-CH pastes, 28d PC represents Portland cement hydrated for 28d.

To better understand the distinct hydration characteristics of LS-CH paste compared with conventional cement systems, Fourier transform infrared spectroscopy (FTIR) was employed to analyze the chemical composition of hydration products. Figure 16 shows the comparative FTIR spectra of: (1) LS-CH paste and (2) Portland cement paste (*w*/*c* = 0.3) after 28 days of hydration under standard curing conditions.

As shown in Figure 16, the distinct band at 3640 cm^−1^, assigned to the stretching vibration modes of H-OH groups, corresponded to calcium hydroxide in both the hardened cement paste and hardened LS-CH pastes. Meanwhile, the band at 875 cm^−1^ is associated with the out-of-plane bending (ν_2_) modes of CO_3_^2−^ and the antisymmetric stretching modes of AlO_4_ groups [53]. This result further confirms that, similar to its role in hardened cement paste, the carbonate phase is also an important hydration product in hardened LS-CH paste, which is consistent with SEM observations.

The main differences between the hydration products of LS-CH pastes and PC paste appeared in the characteristic bands within the ranges of 1000–970 cm^−1^ and 550–520 cm^−1^ [54]. This can be attributed to two factors: firstly, the presence of unreacted spodumene and quartz; secondly, in LS-CH pastes, the band at 970 cm^−1^, which is characteristic of the Si-O stretching vibrations in C–S–H gels, exhibited a significant shift toward higher wavenumbers compared to that in PC paste (from 970 cm^−1^ to 1000 cm^−1^). This phenomenon further indicates an increased content of Al incorporated into C–S–H gels, as well as the gradual polymerization of silicate chains in C–S–H gels accompanied by a decreasing Ca/Si ratio [55]. These findings are consistent with the aforementioned SEM-EDS analysis results.

### 2.7. Hydration Products of LS-CH Pastes Determined by BET Analysis

To further analyze the differences in properties between the hydration products of LS-CH pastes and those of 28-day PC paste, the multipoint Brunauer–Emmett–Teller (BET) analysis was performed to characterize the hydration products of LS-CH paste, with the results presented in Table 5. BET analysis is well suited for characterizing the gel pores in calcium silicate hydrate (C–S–H) and partial transitional pores in hardened LS-CH paste [56]. Accordingly, BET results can provide insights into the structural characteristics of C–S–H to some extent. Specifically, pore structure reflects the compactness of C–S–H, whereas specific surface area acts as an indicator of its cementitious potential.

As shown in Table 5, LS-CH pastes exhibited a coarser pore structure than PC paste, characterized by larger average pore diameters (25.88–43.61 nm vs. 19.09 nm), lower specific surface areas (11.49–17.96 m^2^/g vs. 29.98 m^2^/g), and higher cumulative pore volumes (0.1580–0.1638 cm^3^/g vs. 0.1530 cm^3^/g).

The phase composition of conventional cement hydration products typically consists of 50–60% C–S–H, 20–25% calcium hydroxide, and 5–10% ettringite [57,58]. In contrast, the LS-CH system displayed a markedly distinct phase assemblage: C–S–H content below 24.88% (Figure 10), calcium hydroxide ranging from 5.47% to 7.19% (Table 4), and ettringite between 10.60% and 13.34% (Figure 10). This unique phase composition, differing from that of traditional hardened cement pastes, is the primary cause of the coarser pore structure and lower specific surface area observed in the hardened LS-CH paste. These characteristics are mainly attributed to the low C–S–H content, high ettringite content, and the large proportion of unreacted lithium slag.

In addition, the reticular C–S–H structure formed in the LS-CH paste in this study was relatively loose, with its gel pore size significantly larger than that of C–S–H gel in hardened cement paste [59], reaching several tens of nanometers (Figure 12b, Figure 13a and Figure 14a). Meanwhile, a large number of unreacted lithium slag particles were present in the LS-CH paste. Owing to the inherently porous structure of lithium slag (with an average pore size of 19.96 nm, as shown in Table 5), the incorporation of these unreacted particles further increased the porosity of the paste. Furthermore, it should be noted that although a moderate amount of ettringite can refine the microstructure via pore-filling effects, excessive ettringite formation may instead increase porosity [60].

Extending the curing age promotes the formation of C–S–H (Table 4). Consequently, under both standard and steam curing regimes, prolonged curing (N28d vs. N3d; S7d vs. S7h) yields a finer pore structure, characterized by smaller average pore diameters and larger specific surface areas. This refinement is attributed to the inherently high specific surface area and dense nature of C–S–H gel. In contrast, early-age steam-cured samples (S7h) exhibited larger average pore diameters and specific surface areas than their standard-cured counterparts (N3d). This result arises from a dual effect: while steam curing accelerates early-age C–S–H formation, it also induces rapid hydration that can lead to an uneven distribution of products, thereby introducing more and larger pores. Consistent with this mechanism, S7d samples displayed a larger pore diameter than N28d samples. Nevertheless, owing to the lower total volume of C–S–H formed in S7d samples, their pore volume and specific surface area remained lower than those of N28d.

### 2.8. Mechanical Properties

**Figure 17:**
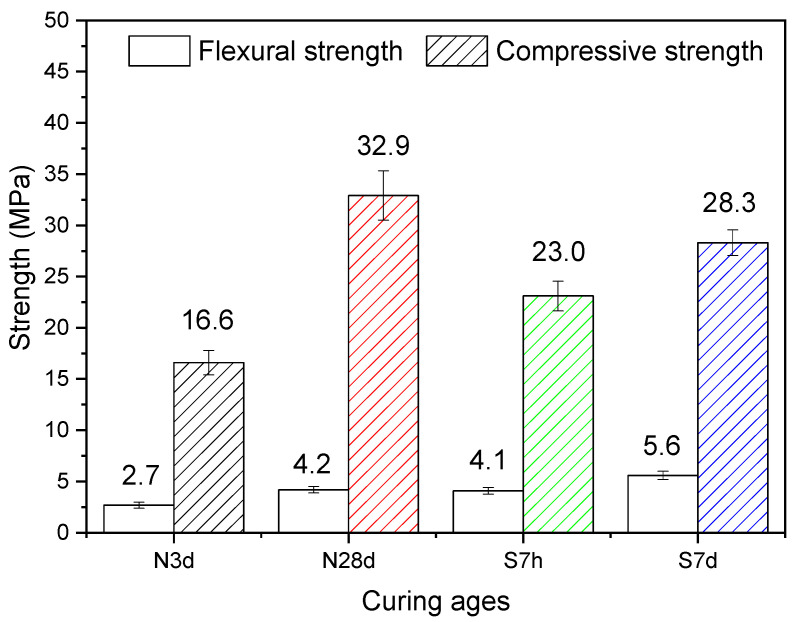
Strength of LS-CH pastes.

Figure 17 presents the mechanical strength development of LS-CH pastes under various curing regimes. Under standard curing conditions, the 3-day compressive and flexural strengths reached 16.6 MPa and 2.7 MPa, respectively, and increased to 32.9 MPa and 4.2 MPa after 28 days of hydration. As discussed earlier, this significant strength development is primarily attributed to the substantial formation of AFt and C–(A)–S–H gels.

Steam-cured specimens exhibited enhanced early-age strength, with 7 h compressive and flexural strengths reaching 23.0 MPa and 4.1 MPa, respectively. These values exceeded those of 3-day normally cured samples. This significant improvement clearly demonstrates the beneficial effect of elevated temperature in activating the hydration potential of lithium slag. However, extending the steam curing duration to 7 days resulted in only moderate strength gains (28.3 MPa compressive and 5.6 MPa flexural), representing increases of just 23.0% and 36.6%, respectively, relative to the 7 h steam-cured specimens.

The compressive strengths of the four specimens—N3d, S7h, S7d, and N28d—increased sequentially. This can be attributed to the gradual rise in the content of newly formed hydration products (Table 4) and the corresponding increase in N_2_ specific surface area (Table 5), which serves as an indicator of C–S–H formation.

Notably, the compressive strength of the 7-day steam-cured specimen (S7d) was lower than that of the 28-day standard-cured specimen (N28d). This behavior is not only associated with the lower quantity of hydration products generated, but also with microstructural defects induced by prolonged high-temperature exposure—especially microcracks (Figure 14a) and a coarsened pore structure (Table 5)—which collectively impaired strength development. Interestingly, however, S7d exhibited a higher flexural strength. This may be attributed to its lower CH content (Table 4). Wang et al. [61] previously reported that a lower content of CH, along with a smaller CH particle size, can improve the interfacial transition zone and thereby help enhance the flexural strength of concrete.

In addition, after 28 days of normal curing, the flexural and compressive strengths of LS-CH are approximately one-third of those of P·I 42.5 cement under the same conditions [62]; whereas under steam curing at 80 °C for 7 days, the compressive strength and flexural strength of LS-CH reach 3.49 times and 1.70 times those of ferronickel slag powder activated by 10% CH, respectively, under identical curing conditions [63]. These favorable mechanical properties not only confirm the high pozzolanic reactivity of lithium slag but also demonstrate the potential of LS-CH paste as a viable cementitious material.

### 2.9. Discussion

#### 2.9.1. Differences Between LS and Traditional SCMs

Distinct from conventional SCMs, lithium slag is characterized by elevated SO_3_ and carbonate contents. These components exist in specific chemical forms: SO_3_ is present mainly as calcium sulfate (both hydrated and anhydrous CaSO_4_) and sodium sulfate (Na_2_SO_4_), while carbonates occur as CaCO_3_, Na_2_CO_3_, and Li_2_CO_3_. SO_3_ plays a crucial role in controlling setting behavior through a dual mechanism: (1) at low concentrations, it retards hydration by moderating C_3_A reactivity, thereby prolonging setting time; (2) at high concentrations, it accelerates hydration through rapid ettringite formation, which may induce flash setting [46,49].

Similarly, the presence of carbonates in LS also exerts a dual effect on the LS-CH system. On the one hand, carbonates react with Ca^2+^ in the system to form CaCO_3_, lowering the Ca^2+^ concentration. This in turn promotes further dissolution of CH and releases more OH^−^, increasing the alkalinity of the system and accelerating the dissolution of the amorphous phase in LS [64]. Meanwhile, carbonate ions can partially replace sulfate ions during ettringite formation, accelerating the setting of the system [40]. On the other hand, the reduced Ca^2+^ concentration retards the nucleation of C–S–H, thereby prolonging the setting time [65].

Although LS is rich in SiO_2_ and Al_2_O_3_ (Table 1), these components mainly exist as crystalline LiAlSi_2_O_6_ rather than in reactive amorphous phases. The low reactivity of LiAlSi_2_O_6_ explains the weaker pozzolanic activity of LS at later ages compared with GBFS, which contains a higher proportion of amorphous aluminosilicate phases. However, high-temperature treatment can significantly improve the reactivity of spodumene (Figure 10 and Figure 13c and Table 4). Therefore, lithium slag is highly suitable for applications in components cured at high temperatures [8,22], or it can be effectively utilized after a high-temperature pretreatment process [66].

#### 2.9.2. Differences Between LS-CH and Traditional Cement Products

Additionally, owing to the high SO_3_ and carbonate contents in lithium slag, ettringite and CaCO_3_ constitute a considerable proportion of the hydration products in hardened LS-CH pastes (Figure 10 and Table 4). Their contents are not only markedly higher than those in conventional cement-based materials but also in some cases even exceed that of C–S–H gel in LS-CH pastes. This distinctive chemical composition exerts several critical influences on the properties of LS-CH paste:

(1) Reduced fluidity and enhanced early strength: The rapid formation of ettringite accelerates paste hardening and improves early-age strength (Figure 17). Meanwhile, it consumes considerable free water, thereby reducing the fluidity of the fresh paste (the fluidity of the LS-CH mortar was 120 mm).
(2) Pore structure: The LS-CH paste displays a coarser pore structure and a smaller specific surface area (Table 5).
(3) Thermal stability of ettringite: Prolonged steam curing provides only limited benefits to the LS-CH paste. Even when the curing duration is increased from 7 h to 7 days, the increases in hydration products and mechanical properties remain negligible, as shown in Table 4 and Figure 15. This limited improvement is mainly controlled by two factors: the thermal instability of ettringite and thermal damage caused by steam curing (Figure 14a), both of which restrict further property development. Therefore, from the perspective of balancing energy costs and strength gain, prolonged high-temperature steam curing should be applied cautiously to LS-CH specimens. It is also worth noting that this limited strength enhancement may be associated with the low CH content in the LS-CH system. Hence, future research could further explore the performance evolution of LS-CH with different CH dosages under various steam curing conditions.
(4) Delayed ettringite formation: The high SO_3_ content raises potential concerns regarding delayed ettringite formation (DEF), which requires further investigation. However, existing literature indicates that in low-alkalinity lime-based systems, carbonation tendencies may suppress the stability of ettringite [67].

#### 2.9.3. Thermal Damage Caused by Steam Curing

As shown in Figure 14a and Table 5, microcracks and pore coarsening appeared in the LS-CH paste under steam curing conditions, indicating that steam curing caused thermal damage to LS-CH.

Thermal damage caused by steam curing to concrete typically includes three types: unrecoverable expansion deformation, surface layer defects, and embrittlement effects, all of which are detrimental to its durability [68]. This can be attributed to three main factors: (1) The rapid and uneven formation of hydration products leads to coarsening and deterioration of the pore structure [69]. (2) Thermal expansion mismatch induces microcracking. At the macroscopic level, the temperature gradient arising from differential heat dissipation between the surface and interior creates thermal stress gradients, resulting in surface microcracks. At the mesoscopic level, the mismatch in thermal expansion coefficients between aggregate and cement paste imposes additional stresses on the interfacial transition zone (ITZ) during heating and cooling. Furthermore, a higher steam-curing temperature leads to a more uneven microhardness distribution in the ITZ and a greater number of interfacial defects [70]. (3) When concrete is heated, the internal free water expands and partially vaporizes, generating internal steam pressure [71].

## 3. Conclusions

To explore the hydration mechanism of lithium slag (LS) in cementitious systems, this study adopts a systematic approach by simulating the alkaline environment of cement using calcium hydroxide (CH). The investigation first compares the hydration processes of the LS-CH system (containing 10 wt.% CH) with those of parallel GBFS-CH and FA-CH systems. Subsequently, the research comprehensively examines the hydration products and mechanical properties of LS-CH paste under both standard curing (3 days and 28 days) and steam curing (80 °C for 7 h and 7 days) conditions. The principal findings can be summarized as follows:

(1) Under CH activation, the reactivity of LS within 72 h is slightly lower than that of GBFS but significantly higher than that of FA. Notably, during the initial 9 h of hydration, the reactivity of LS is superior to that of GBFS, owing to the abundant formation of ettringite in the LS-CH system.
(2) Within 72 h, the hydration heat flow curve of LS-CH exhibits two distinct exothermic peaks. The first peak (0–10 h) corresponds to three simultaneous processes: (I) ettringite formation, (II) dissolution of LS particles in the CH solution, and (III) rapid precipitation of C–S–H gel. In contrast, the secondary peak (30–50 h) arises mainly from calcite crystallization and renewed ettringite formation.
(3) Under standard curing conditions, lithium slag reacts with Ca(OH)_2_ solution to form several typical hydration products: needle-like AFt crystals, spherical/cubic/spindle-shaped CaCO_3_ polymorphs, and loose reticular C–S–H gels. When subjected to 80 °C steam curing, the product assemblage is extended to include AFm phases and calcium sulfate hydrates (both dihydrate and hemihydrate forms). Notably, the persistence of calcium sulfate in the products implies potential risks of delayed ettringite formation (DEF), which deserves careful consideration when using lithium slag as a supplementary cementitious material in concrete.
(4) SEM-EDS and FTIR analyses indicate that the C–S–H gel, the main binding phase in LS-CH, has a unique chemical composition characterized by a low Ca/Si ratio and significant incorporation of aluminum and sulfur. Compared with conventional hardened cement paste, the LS-CH paste shows a larger average pore diameter (25.88–43.61 nm vs. 19.09 nm) and higher pore volume (0.1580–0.1638 cm^3^/g vs. 0.1530 cm^3^/g), but a lower specific surface area (11.49–17.96 m^2^/g vs. 29.98 m^2^/g).
(5) Mechanical tests demonstrate that LS-CH exhibits promising potential as a sustainable alternative to traditional cementitious materials.

## 4. Materials and Methods

### 4.1. Materials

The lithium slag (LS) used in this study was supplied by Jiangsu Rongda New Material Co., Ltd. (Nantong, China).

For comparison, Type S95 ground granulated blast-furnace slag (GBFS) conforming to Chinese National Standard GB/T 18046-2008 [72], Type I fly ash (FA) compliant with Chinese National Standard GB/T 1596-2017 [73], and P·II 52.5 Portland cement (PC) meeting Chinese National Standard GB 175-2007 [74] were employed. It should be clarified that GBFS, FA, and PC were mainly used as reference groups to investigate the hydration process of LS and the characteristics of its hydration products. GBFS and FA were only used in hydration heat tests, whereas PC was solely used to cast specimens with a water-to-cement ratio of 0.3 for FTIR and BET analyses of the 28-day hydration products.

To eliminate the interference of other impurity ions, deionized water and analytically pure calcium hydroxide (Ca(OH)_2_, purity > 95 wt.%) were used to prepare lithium slag-Ca(OH)_2_ pastes.

Standard sand with a fineness modulus of 2.75, conforming to GB/T 17671-2021 [75], was used to prepare LS-CH mortar and perform fluidity test.

### 4.2. Sample Preparation and Test Methods

#### 4.2.1. Sample Preparation

To investigate the mechanism of LS in cement, this study employs CH to simulate the alkaline environment of cement. The hydration process of LS is activated by CH, and the types and characteristics of the hydration products are analyzed to directly reflect the influence of LS on the cement hydration process. Since a CH dosage of 10% has been widely used to activate SCMs [63,76] and to facilitate comparison with existing literature, this study adopts the same CH dosage at the same water-to-binder ratio to activate lithium slag. The detailed mix proportions of the LS–Ca(OH)_2_ mixture (abbreviated as LS–CH) are presented in Table 6. Meanwhile, in accordance with the GB/T 17671-2021 standard [75], three LS–CH prismatic specimens measuring 40 mm × 40 mm × 160 mm were prepared for mechanical property testing.

As shown in Table 6, the setting time of LS-CH, measured in accordance with GB/T 1346-2024 [77], is comparable to that of ordinary Portland cement. The fluidity of the LS-CH mortar was only 120 mm, which can be attributed to the low solubility of calcium hydroxide and the high water demand of lithium slag [78].

For comparison, a paste of PC with a water-to-cement ratio (*w*/*c*) of 0.3 was also prepared and hydrated for 28 days.

**Figure 18:**
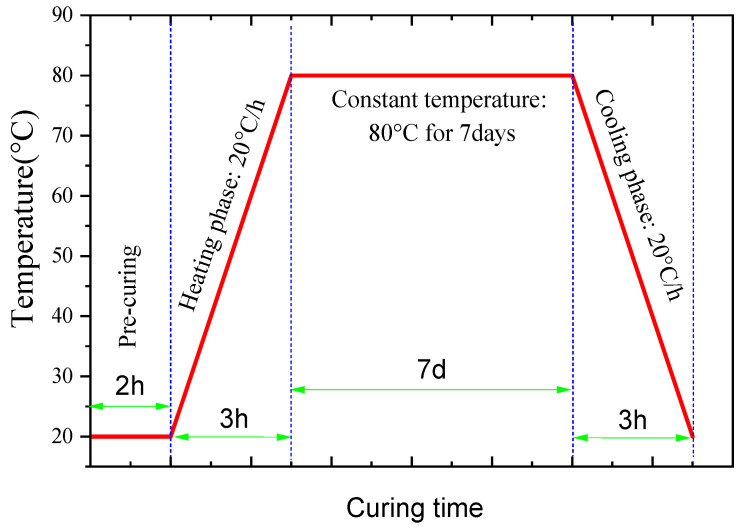
The schematic diagram for steam curing (taking 80 °C for 7 days curing as an example).

To investigate the characteristics of hydration products in LS-CH under different curing conditions, this study employed both normal curing (NC) and steam curing (SC) regimes, as detailed in previous studies [63,79] and illustrated in Figure 18. Specifically, the normal curing regime included two durations: curing at 20 ± 2 °C with a relative humidity (RH) greater than 95% for 3 days and for 28 days, respectively. Meanwhile, the steam curing regime involved constant-temperature curing at 80 °C for 7 h and for 7 days. For clarity, the specimens are designated as follows: N3d and N28d denote LS-CH samples under normal curing for 3 and 28 days, respectively, whereas S7h and S7d represent those subjected to steam curing for 7 h and 7 days.

Compressive strength and flexural strength tests were conducted in accordance with the Chinese standard GB/T 17671-2021 [75].

#### 4.2.2. Test Methods

The setting time of the LS-CH paste was determined using a Vicat apparatus (GB/T 1346-2024 [77]), and its fluidity was tested by the jump table method (GB/T 2419-2024 [80]).

The 28-day pozzolanic activity of SCM was evaluated by the compressive strength ratio of 40 mm × 40 mm × 160 mm prismatic cement mortar specimens. These specimens were prepared with a 30% SCM replacement level and subjected to 28 days of standard curing, with the results compared to those of pure cement specimens.

After the strength tests at the designated ages, hydration of the specimens was stopped by immersion in anhydrous ethanol. The paste samples were then ground into dried powder or cut into approximately 5 mm blocks for characterization by XRD, SEM-EDS, TG-DTG, FTIR, and BET.

X-ray diffraction (XRD) was employed to quantitatively analyze the mineralogical compositions of the LS and LS-CH systems using a Bruker D8-Discover X-ray diffractometer (Karlsruhe, Germany) combined with the Rietveld full-pattern fitting method [8].

The microstructure, phase morphology, and elemental composition of LS and LS-CH were examined using a Sirion field-emission scanning electron microscope (SEM) equipped with an energy-dispersive X-ray spectrometer (EDS) (FEI, Hillsboro, OR, USA).

Thermogravimetric analysis (TG/DTG) and Fourier transform infrared (FTIR) spectroscopy were performed to investigate the phase evolution and structural characteristics of the LS and LS-CH samples, using a STA 449 F3 Jupiter^®^ (Selb, Germany) and a Deaupos Scientific FTIR spectrometer (Berlin, Germany), respectively.

The pore structure parameters and specific surface area of the powdered LS and LS-CH paste were determined from nitrogen adsorption–desorption isotherms using the BET method with a fully automated gas sorption analyzer (ASIQM0001-5, Quantachrome Instruments, Boynton Beach, FL, USA) [81].

Isothermal conduction calorimetry (ICC) test: to clarify the hydration behavior of lithium slag in a CH solution, ICC tests were performed at 20 °C using a TAM Air Calorimeter (Thermometric, Stockholm, Sweden) on pastes with a water-binder ratio of 0.3. To minimize the interference of CH dissolution on LS hydration, CH was pre-dissolved in water in a sealed plastic bottle for 24 h prior to testing. Owing to the small sample mass required for the ICC test, the LS-CH paste was manually mixed at room temperature using a glass rod. For comparison, two commonly used SCMs, namely GBFS and FA, were selected as reference materials. The hydration processes of GBFS-CH and FA-CH pastes were also tested under identical conditions.

Furthermore, to ensure the reliability and reproducibility of the experimental results, all tests were performed in duplicate or triplicate.

## Figures and Tables

**Table 1:** The chemical compositions of LS, GBFS, FA and Portland cement (PC), wt.%.

Materials	CaO	SiO_2_	Al_2_O_3_	SO_3_	Fe_2_O_3_	MgO	P_2_O_5_	Na_2_O
LS	4.53	62.40	22.1	6.73	1.06	0.49	1.12	0.89
GBFS	36.05	34.67	16.52	2.53	0.29	3.90	4.95	-
FA	6.56	54.71	26.74	1.42	5.00	1.50	1.44	-
PC	64.47	20.87	4.87	2.52	3.59	2.13	-	0.11

**Table 2:** Atomic composition of particles 1~5 in Figure 3, at.%.

Particles	Ca	Si	Al	O	S	Mg
1	0.61	32.21	13.91	52.69	0.58	
2	0.47	26.00	11.58	61.96		
3		24.38	11.30	63.83	0.49	
4	15.43	13.55	7.13	58.73	1.30	3.86
5	9.79	9.89	5.40	66.88	8.05	

**Table 3:** Reactions occur within specific temperature ranges [27,28,29,51,52].

Temperature Ranges	Reactions
60–150 °C	The loss of bound water from the decomposition of gypsum, ettringite, and C–S–H
150–400 °C	The decomposition of monosulfates, aluminate hydrates, hydrogarnet series phases, and calcium aluminosilicate hydrates, e.g., gehlenite hydrate, stratlingite, etc.
400–550 °C	Dehydroxylation of CH
550–1000 °C	Decarbonation of carbonates

**Table 4:** The mass loss of LS-CH pastes determined by TG analysis within specified temperature ranges, along with the CH content (wt.%).

Sample	60–150 °C	150–400 °C	400–550 °C	550–1000 °C	Total Mass Loss	CH
S7h	4.93	1.75	1.36	2.12	10.16	5.59
N3d	4.56	1.83	1.75	2.32	10.46	7.19
S7d	4.98	2.30	1.33	2.32	10.93	5.47
N28d	5.38	2.35	1.68	2.31	11.72	6.90

**Table 5:** BET results of LS-CH pastes.

Sample	Average Pore Width (nm)	Cumulative Pore Volume (cm^3^/g)	N_2_-Specific Surface Area (m^2^/g)
LS	19.96	0.0285	4.400
S7h	43.61	0.1631	11.94
N3d	38.01	0.1638	11.49
S7d	40.49	0.1580	12.39
N28d	25.88	0.1663	17.96
28d PC	19.09	0.1530	29.98

**Table 6:** Mix proportion (wt.%), setting time and fluidity of LS-CH pastes.

LS	CH	Water	Water/(LS + CH) Ratio	Setting Time (h: min)		Fluidity (mm)
				Initial	Final	
90	10	30	0.3	1:06	2:25	120

## Data Availability

All data generated or analyzed during this study are included in this submitted article.

## Abbreviations

The following abbreviations are used in this manuscript:

LS	Lithium slag
SCM	Supplementary cementitious material
GBFS	Ground granulated blast-furnace slag
FA	Fly ash
PC	Ordinary Portland cement
ASTM	American Society for Testing and Materials
AAMs	Alkali-activated materials
C−S−H	Calcium silicate hydrate
CH	Calcium hydroxide
AFt	Ettringite
AFm	Monosulfoaluminate
XRF	X-ray fluorescence
LOI	Loss on ignition
XRD	X-ray diffractometer
SEM-EDS	Scanning electron microscope coupled with an X-ray energy-dispersive spectrometer
TG-DTG	Thermogravimetric and derivative thermogravimetric analyses
FTIR	Fourier transform infrared spectroscopy
BET	Brunauer–Emmett–Teller (BET) analysis
ICC	Isothermal conduction calorimetry test
ITZ	Interfacial transition zone

## References

[1] Amin M.T.E., Sarker P.K., Shaikh F.U.A. (2024). Transport properties of concrete containing lithium slag. Constr. Build. Mater..

[2] Yang H., Wang B., Lin R.S. (2025). Workability, hydration, and carbonation of cementitious materials with high-volume of lithium slag/steel slag: A comparative study with limestone. Constr. Build. Mater..

[3] Zhai J., Chen P., Long J., Fan C., Chen Z., Sun W. (2024). Recent advances on beneficial management of lithium refinery residue in China. Miner. Eng..

[4] Wang Y., Wang D., Cui Y., Zheng D., Liu Z. (2019). Micro-morphology and phase composition of lithium slag from lithium carbonate production by sulphuric acid process. Constr. Build. Mater..

[5] Zhang L. (2007). Experiment study on high-performance lithium-slag concrete. J. Liaoning Tech. Univ..

[6] Chen D., Hu X., Shi L., Cui Q., Wang H., Yao H. (2012). Synthesis and characterization of zeolite X from lithium slag. Appl. Clay Sci..

[7] Tan H., Li X., He C., Ma B., Bai Y., Luo Z. (2015). Utilization of lithium slag as an admixture in blended cements: Physico-mechanical and hydration characteristics. J. Wuhan Univ. Technol.-Mater. Sci. Ed..

[8] Li B., Cao R., You N., Chen C., Zhang Y. (2019). Products and properties of steam cured cement mortar containing lithium slag under partial immersion in sulfate solution. Constr. Build. Mater..

[9] Lin G., Zhuang Q., Cui Q., Wang H., Yao H. (2015). Synthesis and adsorption property of zeolite FAU/LTA from lithium slag with utilization of mother liquid. Chin. J. Chem. Eng..

[10] Ding J., Bai Y., Cai Y. (2008). Suppressing effect of lithium slag on alkali-silica reaction and separation of its self-expansion. J. Hohai Univ. (Nat. Sci.).

[11] He Y., Chen Q., Qi C., Zhang Q., Xiao C. (2019). Lithium slag and fly ash-based binder for cemented fine tailings backfill. J. Environ. Manag..

[12] He Y., Zhang Q., Chen Q., Bian J., Qi C., Kang Q., Feng Y. (2021). Mechanical and environmental characteristics of cemented paste backfill containing lithium slag-blended binder. Constr. Build. Mater..

[13] Li J., Huang S. (2020). Recycling of lithium slag as a green admixture for white reactive powder concrete. J. Mater. Cycles Waste Manag..

[14] Li J., Lian P., Huang S., Huang L. (2020). Recycling of lithium slag extracted from lithium mica by preparing white Portland cement. J. Environ. Manag..

[15] Zhou X., Tang Z., Zheng Y., Zhang Y., Wu F. (2025). Research on the properties and mechanism of a fiber-reinforced alkali-activated lithium slag artificial lightweight aggregate. Constr. Build. Mater..

[16] Escalante-García J.I., Sharp J.H. (1998). Effect of temperature on the hydration of the main clinker phases in Portland cements: Part II, blended cements. Cem. Concr. Res..

[17] Rahman S.A., Dodd A., Khair S., Shaikh F.U.A., Sarker P.K., Hosan A. (2023). Assessment of lithium slag as a supplementary cementitious material: Pozzolanic activity and microstructure development. Cem. Concr. Compos..

[18] Liu Z., Wang J., Jiang Q., Cheng G., Li L., Kang Y., Wang D. (2019). A green route to sustainable alkali-activated materials by heat and chemical activation of lithium slag. J. Clean. Prod..

[19] Tan H., Zhang X., He X., Guo Y., Deng X., Su Y., Wang Y. (2018). Utilization of lithium slag by wet-grinding process to improve the early strength of sulphoaluminate cement paste. J. Clean. Prod..

[20] Tan H., Li M., He X., Su Y., Yang J., Zhao H. (2021). Effect of wet grinded lithium slag on compressive strength and hydration of sulphoaluminate cement system. Constr. Build. Mater..

[21] Khair S., Rahman S.A., Shaikh F.U.A., Sarker P.K. (2024). Evaluating lithium slag for geopolymer concrete: A review of its properties and sustainable construction applications. Case Stud. Constr. Mater..

[22] Li B., Wail S., Shi L., Arif A., Huo B., Cheng Y. (2026). The Early Age Hydration Products and Mechanical Properties of Autoclaved Cement Paste Incorporating Supplementary Cementitious Materials. Gels.

[23] Hewlett P.C. (1998). Lea’s Chemistry of Cement and Concrete.

[24] He Z., Li L., Du S. (2017). Mechanical properties, drying shrinkage, and creep of concrete containing lithium slag. Constr. Build. Mater..

[25] He Z., Du S., Chen D. (2018). Microstructure of ultrahigh performance concrete containing lithium slag. J. Hazard. Mater..

[26] Botto I.L. (1985). Structural and spectroscopic properties of leached spodumene in the acid roast processing. Mater. Chem. Phys..

[27] Hudson-Lamb D.L., Strydom C.A., Potgieter J.H. (1996). The thermal dehydration of natural gypsum and pure calcium sulphate dihydrate (gypsum). Thermochim. Acta.

[28] Strydom C.A., Hudson-Lamb D.L., Potgieter J.H., Dagg E. (1995). The thermal dehydration of synthetic gypsum. Thermochim. Acta.

[29] Alarcon-Ruiz L., Platret G., Massieu E., Ehrlacher A. (2005). The use of thermal analysis in assessing the effect of temperature on a cement paste. Cem. Concr. Res..

[30] Wu M., Sui S., Zhang Y., Jia Y., She W., Liu Z., Yang Y. (2021). Analyzing the filler and activity effect of fly ash and slag on the early hydration of blended cement based on calorimetric test. Constr. Build. Mater..

[31] Biernacki J.J., Richardson J.M., Stutzman P.E., Bentz D.P. (2002). Kinetics of slag hydration in the presence of calcium hydroxide. J. Am. Ceram. Soc..

[32] Scrivener K.L., Juilland P., Monteiro P.J.M. (2015). Advances in understanding hydration of portland cement. Cem. Concr. Res..

[33] Pourchet S., Regnaud L., Perez J.P., Nonat A. (2009). Early C_3_A hydration in the presence of different kinds of calcium sulfate. Cem. Concr. Res..

[34] Minard H., Garrault S., Regnaud L., Nonat A. (2007). Mechanisms and parameters controlling the tricalcium aluminate reactivity in the presence of gypsum. Cem. Concr. Res..

[35] Chan S.Y., Ji X. (1999). Comparative study of the initial surface absorption and chloride diffusion of high performance zeolite, silica fume and PFA concretes. Cem. Concr. Compos..

[36] Siddique R., Klaus J. (2009). Influence of metakaolin on the properties of mortar and concrete: A review. Appl. Clay Sci..

[37] Sabir B.B., Wild S., Bai J. (2001). Metakaolin and calcined clays as pozzolans for concrete: A review. Cem. Concr. Compos..

[38] Ramezanianpour A.A. (2014). Cement Replacement Materials.

[39] Borges P.H., Costa J.O., Milestone N.B., Lynsdale C.J., Streatfield R.E. (2010). Carbonation of CH and C–S–H in composite cement pastes containing high amounts of BFS. Cem. Concr. Res..

[40] Taylor H.F.W. (1997). Cement Chemistry.

[41] Yu Q., Sawayama K., Sugita S., Shoya M., Isojima Y. (1999). The reaction between rice husk ash and Ca(OH)_2_ solution and the nature of its product. Cem. Concr. Res..

[42] Faucon P., Adenot F., Jacquinot J.F., Petit J.C., Cabrillac R., Jorda M. (1998). Long-term behaviour of cement pastes used for nuclear waste disposal: Review of physico-chemical mechanisms of water degradation. Cem. Concr. Res..

[43] Taylor H.F.W., Famy C., Scrivener K.L. (2001). Delayed ettringite formation. Cem. Concr. Res..

[44] Siedel H., Hempel S., Hempel R. (1993). Secondary ettringite formation in heat treated Portland cement concrete: Influence of different W/C ratios and heat treatment temperatures. Cem. Concr. Res..

[45] Hall C., Barnes P., Billimore A.D., Jupe A.C., Turrillas X. (1996). Thermal decomposition of ettringite Ca_6_[Al(OH)_6_]_2_(SO_4_)_3_·26H_2_O. J. Chem. Soc. Faraday Trans..

[46] Andrade Neto J.S., Angeles G., Kirchheim A.P. (2021). Effects of sulfates on the hydration of Portland cement—A review. Constr. Build. Mater..

[47] Han Y.S., Hadiko G., Fuji M., Takahashi M. (2005). Effect of flow rate and CO_2_ content on the phase and morphology of CaCO_3_ prepared by bubbling method. J. Cryst. Growth.

[48] Manoli F., Dalas E. (2000). Spontaneous precipitation of calcium carbonate in the presence of ethanol, isopropanol and di-ethylene glycol. J. Cryst. Growth.

[49] Bérodier E.M.J., Muller A.C.A., Scrivener K.L. (2020). Effect of sulfate on C-S-H at early age. Cem. Concr. Res..

[50] Adu-Amankwah S., Black L., Skocek J., Ben Haha M., Zajac M. (2018). Effect of sulfate additions on hydration and performance of ternary slag-limestone composite cements. Constr. Build. Mater..

[51] Palou M.T., Kuzielová E., Žemlička M., Boháč M., Novotný R. (2016). The effect of curing temperature on the hydration of bi-nary Portland cement. J. Therm. Anal. Calorim..

[52] Palou M., Kozánková J., Ifka T. (2010). Determination of activation effect of Ca(OH)_2_ upon the hydration of BFS and related heat by isothermal calorimeter. J. Therm. Anal. Calorim..

[53] Ismail I., Bernal S.A., Provis J.L., San Nicolas R., Hamdan S., van Deventer J.S.J. (2014). Modification of phase evolution in alkali-activated blast furnace slag by the incorporation of fly ash. Cem. Concr. Compos..

[54] Naskar M.K., Chatterjee M. (2005). A novel process for the synthesis of lithium aluminum silicate powders from rice husk ash and other water-based precursor materials. Mater. Lett..

[55] Huang Y., Wang Q., Shi M. (2017). Characteristics and reactivity of ferronickel slag powder. Constr. Build. Mater..

[56] de Burgh J.M., Foster S.J., Valipour H.R. (2016). Prediction of water vapour sorption isotherms and microstructure of hardened Portland cement pastes. Cem. Concr. Res..

[57] Jiménez Segura N., Pichler B.L.A., Hellmich C. (2023). Mix-, storage- and temperature-invariant precipitation characteristics in white cement paste, expressed through an NMR-based analytical model. Cem. Concr. Res..

[58] Meng Z., Liu Q., Ukrainczyk N., Mu S., Zhang Y., De Schutter G. (2024). Numerical study on the chemical and electrochemical coupling mechanisms for concrete under combined chloride-sulfate attack. Cem. Concr. Res..

[59] Muller A.C.A., Scrivener K.L. (2017). A reassessment of mercury intrusion porosimetry by comparison with 1H NMR relaxometry. Cem. Concr. Res..

[60] Yang Y., Zhan B., Wang J., Zhang Y., Duan W. (2020). Damage evolution of cement mortar with high volume slag exposed to sulfate attack. Constr. Build. Mater..

[61] Wang Y., Zeng D., Ueda T., Fan Y., Li C., Li J. (2021). Beneficial Effect of Nanomaterials on the Interfacial Transition Zone (ITZ) of Non-Dispersible Underwater Concrete. Constr. Build. Mater..

[62] Huang F., Hu Z., Li H., Wang Y., Liu J. (2023). Deformation Mechanisms of Cement Paste with Ultra-Low Water-to-Cement Ratios Under Different Curing Conditions at Early Ages. Constr. Build. Mater..

[63] Li Y., Li B., Yu H., Wail S., Huo B., Cheng Y., Liu Z. (2026). Activation Potential of Various Activators for Ferronickel Slag Under Steam Curing: Characterization of Hydration Products and Mechanical Properties. Gels.

[64] Wang J., Lyu X., Wang L., Cao X., Liu Q., Zang H. (2018). Influence of the Combination of Calcium Oxide and Sodium Carbonate on the Hydration Reactivity of Alkali-Activated Slag Binders. J. Clean. Prod..

[65] Yuan B., Wang H., Chen W., Yu Q. (2024). Solubility-Controlled Early Age Hydration of Carbonate-Activated Slag: Underlying Mechanisms and Acceleration via Seeding. Constr. Build. Mater..

[66] Javed U., Shaikh F.U.A., Sarker P.K. (2022). Microstructural investigation of thermo-mechanically processed lithium slag for geopolymer precursor using various characterization techniques. Constr. Build. Mater..

[67] Nishikawa T., Suzuki K., Ito S., Sato K., Takebe T. (1992). Decomposition of synthesized ettringite by carbonation. Cem. Concr. Res..

[68] Xiang Y., Long G., Xie Y., Zheng K., He Z., Ma K., Zeng X., Wang M. (2020). Thermal Damage and Its Controlling Methods of High-Speed Railway Steam-Cured Concrete: A Review. Struct. Concr..

[69] Cai L., Tian X., Wang H., Deng X., Ge X., Zhang Y., Li D. (2026). Mechanical Performance and Hydration Behavior of Anhydrite Blended Concrete Under Different Steam Curing Regimes. Case Stud. Constr. Mater..

[70] Wang M., Xie Y., Long G., Ma C., Zeng X. (2019). Microhardness Characteristics of High-Strength Cement Paste and Interfacial Transition Zone at Different Curing Regimes. Constr. Build. Mater..

[71] Shi J., Liu B., Wu X., Qin J., Jiang J., He Z. (2020). Evolution of Mechanical Properties and Permeability of Concrete During Steam Curing Process. J. Build. Eng..

[72] (2018). Code for Ground Granulated Blast Furnace Slag Used for Cement, Mortar and Concrete.

[73] (2017). Code for Fly Ash Used for Cement and Concrete.

[74] (2024). Code for Common Portland Cement.

[75] (2021). Code for Test method of Cement Mortar Strength (ISO Method).

[76] Zhu X., Tang D., Yang K., Zhang Z., Li Q., Pan Q., Yang C. (2018). Effect of Ca (OH)_2_ on shrinkage characteristics and microstructures of alkali-activated slag concrete. Constr. Build. Mater..

[77] (2024). Code for Test Methods for Water Requirement of Normal Consistency, Setting Time and Soundness of the Portland Cement.

[78] Euler W.B., Kirschenbaum L.J., Ruekberg B. (2000). Determination of Ksp, ΔG0, ΔH0, and ΔS0. J. Chem. Educ..

[79] Li B., Tang Z., Huo B., Liu Z., Cheng Y., Ding B., Zhang P. (2022). The Early Age Hydration Products and Mechanical Properties of Cement Paste Containing GBFS under Steam Curing Condition. Buildings.

[80] (2017). Code for Test Method for Fluidity of Cement Mortar.

[81] You N., Li B., Cao R., Shi J., Chen C., Zhang Y. (2019). The influence of steel slag and ferronickel slag on the properties of alkali-activated slag mortar. Constr. Build. Mater..

